# Folate Metabolism Regulates Oligodendrocyte Survival and Differentiation by Modulating AMPKα Activity

**DOI:** 10.1038/s41598-017-01732-1

**Published:** 2017-05-11

**Authors:** Qinjie Weng, Jiajia Wang, Jiaying Wang, Biqin Tan, Jing Wang, Haibo Wang, Tao Zheng, Q. Richard Lu, Bo Yang, Qiaojun He

**Affiliations:** 1 Institute of Pharmacology & Toxicology, Zhejiang Province Key Laboratory of Anti-Cancer Drug Research, College of Pharmaceutical Sciences, Zhejiang University, Hangzhou, China; 2 Center for drug safety Evaluation and Research, Zhejiang University, Hangzhou, China; 3 Department of Pediatrics, Brain Tumor Center, Cancer and Blood Disease Institute, Cincinnati Children’s Hospital Medical Center, OH, USA; 4 School of Preclinical and Forensic Medicine, West China Second Hospital, Sichuan University, Chengdu, China

## Abstract

Folate, an essential micronutrient, is a critical cofactor in one-carbon metabolism for many cellular pathways including DNA synthesis, metabolism and maintenance. Folate deficiency has been associated with an increased risk of neurological disease, cancer and cognitive dysfunction. Dihydrofolate reductase (DHFR) is a key enzyme to regulate folate metabolism, however folate/DHFR activity in oligodendrocyte development has not been fully understood. Here we show that folate enhances oligodendrocyte maturation both *in vitro* and *in vivo*, which is accompanied with upregulation of oligodendrocyte-specific DHFR expression. On the other hand, pharmacological inhibition of DHFR by methotrexate (MTX) causes severe defects in oligodendrocyte survival and differentiation, which could be reversed by folate intake. We further demonstrate that folate activates a metabolic regulator AMPKα to promote oligodendrocyte survival and differentiation. Moreover, activation of AMPKα partially rescues oligodendrocyte defects caused by DHFR-inhibition both *in vitro* and *in vivo*. Taken together, these findings identify a previously uncharacterized role of folate/DHFR/AMPKα axis in regulating oligodendrocyte survival and myelination during CNS development.

## Introduction

Myelination by oligodendrocytes in the central nervous system (CNS) ensures saltatory nerve conduction and is essential for brain function. Failure of myelination by oligodendrocytes disrupts the conduction velocity of nerve impulses, leading to nerve degeneration associated with acquired and inherited disorders such as multiple sclerosis (MS) and leukodystrophies^1, 2^. At present, the mechanisms promoting CNS myelination are not fully understood^3^.

Folate, acting as a coenzyme for cellular one carbon metabolism, is essential for the synthesis of DNA and the metabolism of amino acids such as homocysteine, methionine and glycine^4^. The genetic mutations of folate metabolism-related genes, such as dihydrofolate reductase (DHFR) and folate receptor α (FRα), have presented profound folate deficiency^5–8^. Folate gene polymorphisms or folate deficiency in the CNS is a condition described recently, which is associated with various neurological diseases, such as neural tube defects (NTDs), stroke, Parkinson’s disease, and dementia^9–11^. However, there is no direct evidence to show the correlation between MS pathogenesis or oligodendrocyte myelination and folate^12, 13^. A clinical study indicates folate receptor 1 (Folr1) mutations impair cerebral folate transport and cause white matter hypomyelination^14^. Folate deficiency causes abnormal ratio of myelin lipid composition in the brain of developing rats^15^, and is associated with impaired lipid metabolism due to altered methylation^16^, however, the function of folate during myelination is not fully understood. In addition, recent studies indicate that high homocysteine levels may contribute to the pathogenesis of MS^17^. Though folate acts to accelerate the degradation of homocysteine, its role in MS pathology (oligodendrocyte loss and demyelination) remains controversial^18, 19^. Thus, understanding the effect of folate supplement and folate metabolic pathway activation on oligodendrocyte development is important for devising remyelination strategies in the CNS.

In folate metabolism, one-carbon groups are transferred from folate to the synthesis of purines and thymidylates^20^, characterized by adenosine monophosphate (AMP). Folate deficiency causes the downregulation of AMP, leading to inhibition of AMP-activated protein kinase (AMPK) activity indirectly^21, 22^. AMPK is a heterotrimeric, multi-substrate kinase composed of α (catalytic), β (regulatory), and γ (AMP/ATP binding) subunits, which plays an important role in cellular energy homeostasis. Dietary folate supplement restores AMPK activation in high-fat diet mice^23^. Furthermore, recent studies show that AMPK not only affects metabolism, but also regulates growth and differentiation of myelinating cells^24–26^. Loss-of-function of either AMPK subunits causes severe neurological defects including progressive neurodegeneration and severe hypomyelination^27^. On the other hand, AMPK signaling activation effectively protects oligodendrocytes through immune modulation in experimental autoimmune encephalomyelitis (EAE), an animal model of MS^28, 29^. Based on these results, we hypothesize that folate-regulation of AMPK activity is required for CNS myelination.

In this study, we investigate the role of folate in the process of oligodendrocyte myelination. We find that folate supplement during pregnancy and lactation accelerates oligodendrocyte maturation by activating DHFR, a key enzyme in folate metabolism. Conversely, pharmacological DHFR inhibition by methotrexate (MTX) leads to oligodendrocyte death and differentiation defects, which can be rescued by folate supplement. Folate/DHFR signaling pathway could enhance the phosphorylation of AMPKα to promote oligodendrocyte myelination. Together, these findings indicate a novel role of folate/DHFR/AMPKα signaling axis in regulating oligodendrocyte development.

## Results

### Folate is required for the development of oligodendrocytes

**Figure 1:**
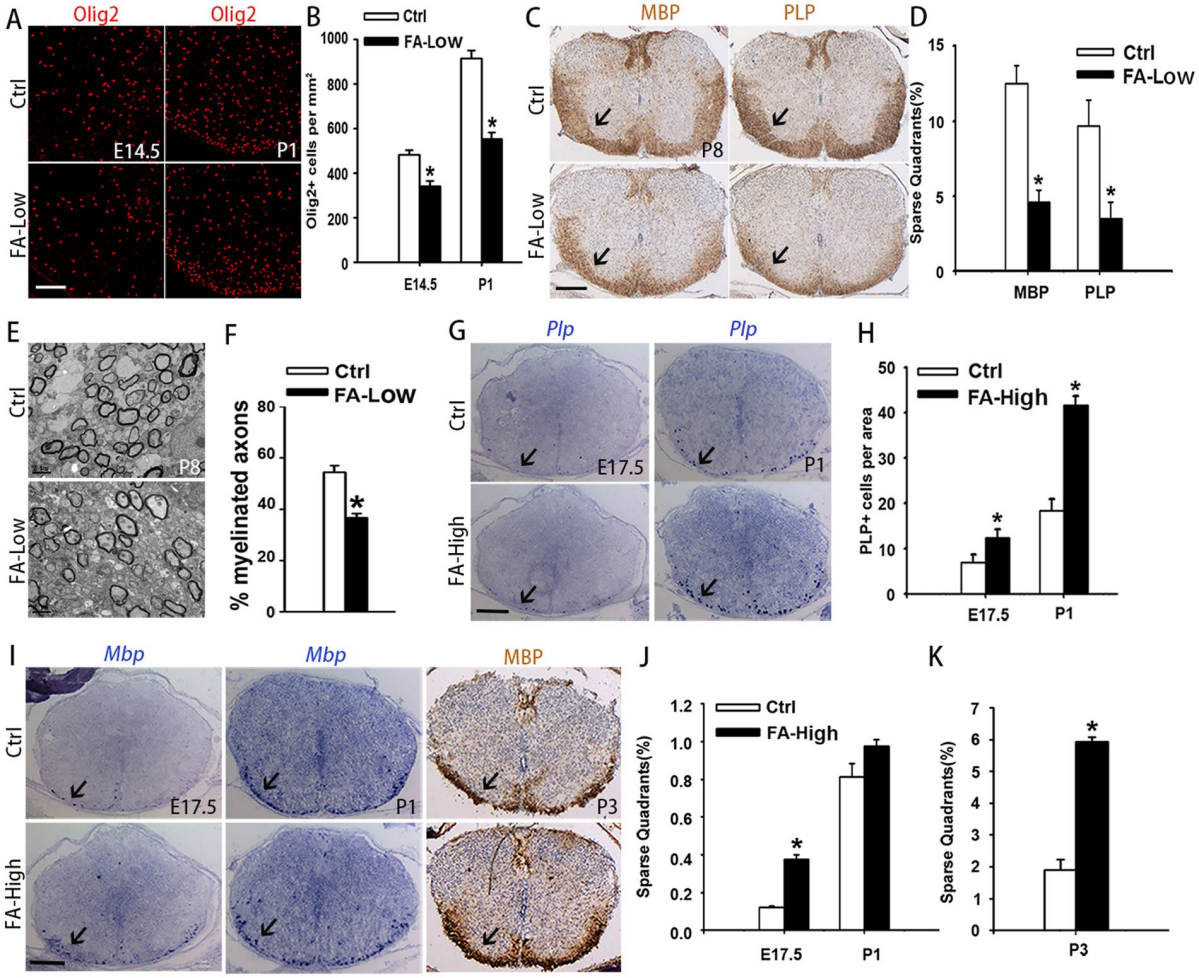
Folate promotes the development of oligodendrocytes *in vivo*. (**A**) Spinal cord isolated from control and FA-Low mice stained with Olig2 antibody at E14.5 and P1. The ventral parts showed in the images. (**B**) Quantification of the numbers of Olig2+ cells per mm^2^ in ventral part of spinal cords of (**A**) is shown in (**B**). Data represents the mean ± S.D. (n = 5, *p < 0.05 compared with control, student’s t test). (**C**) Immunohistochemistry analysis of spinal cords isolated from normal folate-supplemented diet (2 mg folate/kg diet, Ctrl) or low folate-supplemented diet (0.2 mg folate/kg diet, FA-Low) mice stained with MBP and PLP antibodies at P8. (**D**) The number of quadrants with sparse staining for MBP and PLP of (**C**) scored and expressed as a percentage of the total number of quadrants. (n = 5, *p < 0.05 compared with control, student’s t test). (**E**,**F**) Electron micrographs of spinal cords from control and FA-Low mice at P8. (**F**) Shows the percentage of myelinated axons. Data represent the mean ± S.E.M. from three animals (*p < *0*.*05* compared with control, student’s t test). (**G**,**I**) *In situ* hybridization and immunohistochemistry analysis of PLP and MBP on the Spinal cord isolated from E17.5, P1, P3 control and FA-High mice. Arrows point to the white matter. (**H**) Quantification of the numbers of PLP + cells per area (one section of spinal cord) in spinal cords of (**G**) is shown in (**H**). Data represent the mean ± S.D. (n = 5, *p < *0*.*05* compared with control, student’s t test). (**J**,**K**) The number of quadrants with sparse staining for MBP of (**I**) scored and expressed as a percentage of the total number of quadrants. (n = 5, *p < *0*.*05* compared with control, student’s t test). Scale bars: 100 μm (**A**), 200 μm (**C,G,I**), 2 μm (**E**).

Folate deficiency causes neural tube defects (NTDs)^30, 31^, however, little is known about the role of folate in myelination in the CNS. We used a modified version of a previously established folate-deficient mouse model to investigate its role on oligodendrocyte development^32, 33, 34^. Briefly, mice received normal folate-supplemented diet (AIN-93G, 2 mg folate/kg diet, Ctrl) or low folate-supplemented diet (0.2 mg folate/kg diet, FA-Low) from 10 days prior to mating^35^. Spinal cords and brains from different stages of mice including embryos and pups were collected and analyzed. Immunostaining of Olig2 (oligodendrocyte lineage marker) in the spinal white matter from FA-Low and control mice at E14.5 and P1 indicated that FA-Low diet decreased the number of Olig2-positive cells significantly compared with the control (Fig. 1A,B). This suggests that low folate intake may cause oligodendrocyte differentiation defects. Furthermore, expression of myelin basic protein (MBP) and myelin proteolipid protein (PLP) expression was reduced significantly in the spinal cord of FA-Low mice in contrast with control mice at P8 (Fig. 1C,D). Consistently, electron microscopy analysis showed that FA-Low diet resulted in a reduction of the percentage of myelinated axons at P8 (Fig. 1E,F). NeuN (a neuronal marker) expression appeared comparable in spinal cords or brains between FA-Low and control mice (Fig. S1), suggesting that low folate intake may cause apparent oligodendrocyte differentiation defects ahead of neuron loss. In contrast, high folate-supplemented diet (40 mg folate/kg diet, FA-High) increased the number of *Plp* ^+^ or *Mbp* ^+^ cells in spinal cords of mice at E17.5, P1 and P3 assayed by *in situ* hybridization and immunohistochemistry analysis (Fig. 1G–K). This indicates that folate supplement facilitates oligodendrocyte differentiation during pregnancy.

### Folate activates DHFR expression and promotes oligodendrocyte maturation *in vitro*

**Figure 2:**
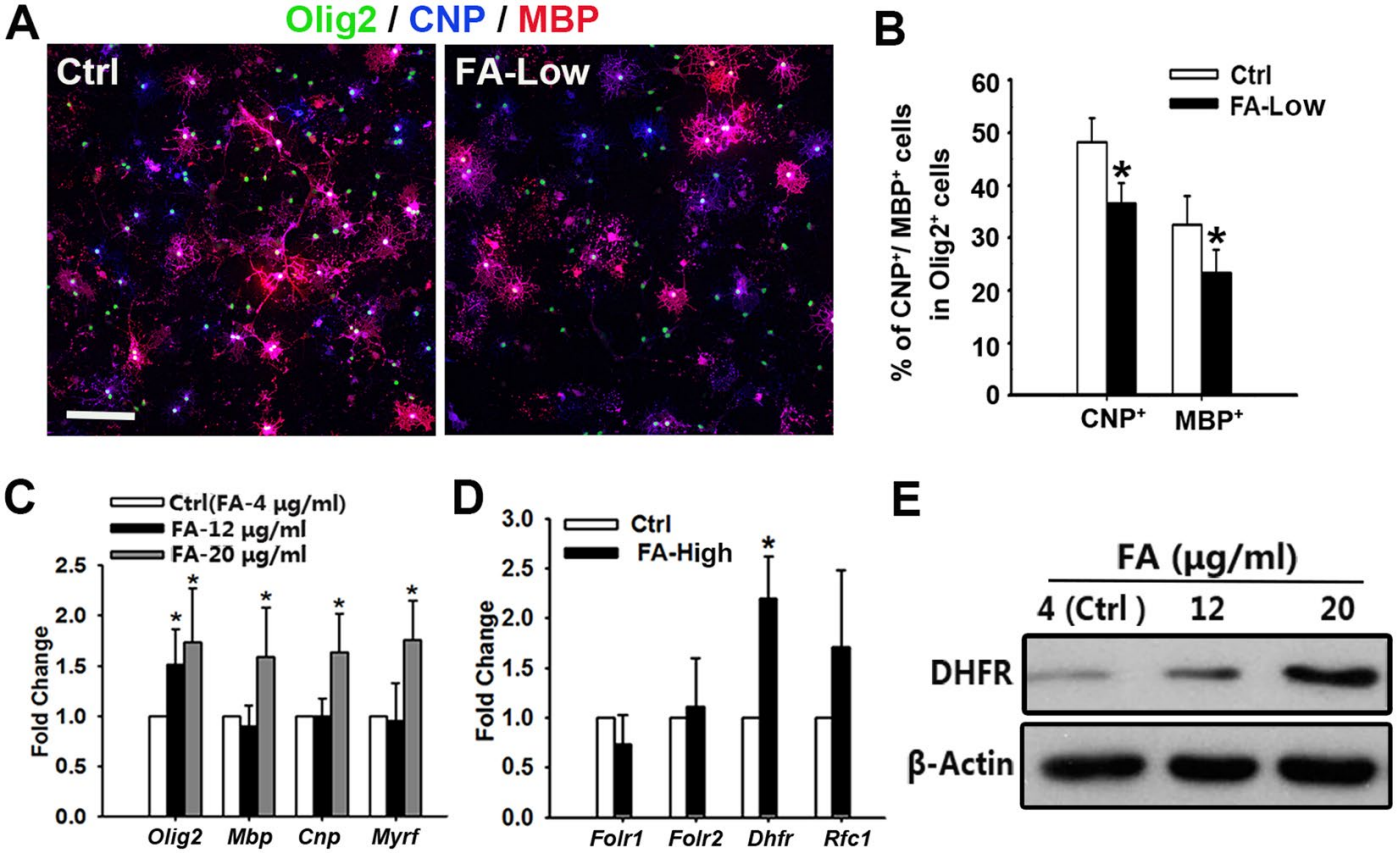
Folate promotes oligodendrocyte maturation depending on DHFR activation *in vitro*. (**A**) Rat cortical oligodendrocyte precursors (OPCs) from pups at P2 isolated and cultured with different concentrations of folate (Ctrl: folate 4 μg/ml, FA-Low: folate 0.02 μg/ml) for 3 days stained with Olig2, CNP and MBP antibodies. (**B**) Quantification of the percentage of CNP^+^ or MBP^+^ cells among Olig2^+^ cells in (**A**) are shown in (**B**). Data represents the mean ± S.D. (n = 5, *p < *0*.*05*, student’s t test). (**C**) qRT-PCR analysis of oligodendrocyte and myelin associated genes and proteins expression after folate treatment for 48 h in Oli-neu cells. Data represents the mean ± S.D. (n > 3, *p < *0*.*05* compared with control, student’s t test). (**D**) qRT-PCR analysis of folate metabolism associated genes expression from spinal cords of control and FA-High mice at P3. Data represents the mean ± S.D. (n > 3, *p < *0*.*05* compared with control, student’s t test). (**E**) Western blot analysis of DHFR (20 kDa) expression in the spinal cord of control and MTX (4 mg/kg)-treated mice. β-actin (43 kDa) is the loading control. Scale bars: 100 μm (**A**).

To further confirm whether folate alone is required for oligodendrocyte maturation, primary oligodendrocyte precursor cells (OPCs) were isolated from neonatal rat brain at P2 and cultured in oligodendrocyte growth medium containing the mitogen PDGF-AA and different concentrations of folate (FA-Low: 0.02 μg/ml, Ctrl: 4 μg/ml) for 3 days and then co-stained with antibodies to MBP, CNP and Olig2. Compared to control, FA-Low resulted in notable decrease in expression of myelin protein CNP and MBP (Fig. 2A,B). In contrast, folate supplement significantly increased the expression of *Olig2*, *Mbp*, *Cnp* and *Myrf* genes in Oli-neu cells, an oligodendrocyte cell line, in a dose-dependent way (Fig. 2C). We further examined the folate metabolism-related genes expression in spinal cords of dietary folate-fed mice. *Dhfr* mRNA level was upregulated significantly in FA-High mice compared with the control, while folate receptor 1 (Folr1), folate receptor 2 (Folr2) and reduced folate carrier 1 (Rfc1) were not changed (Fig. 2D). Western blotting also showed that expression of DHFR increased after folate treatment (Fig. 2E). These data indicate that folate supplement activates DFHR expression and promote OPC maturation.

### Folate-activated DHFR is highly expressed in oligodendrocytes and required for oligodendrocyte development

**Figure 3:**
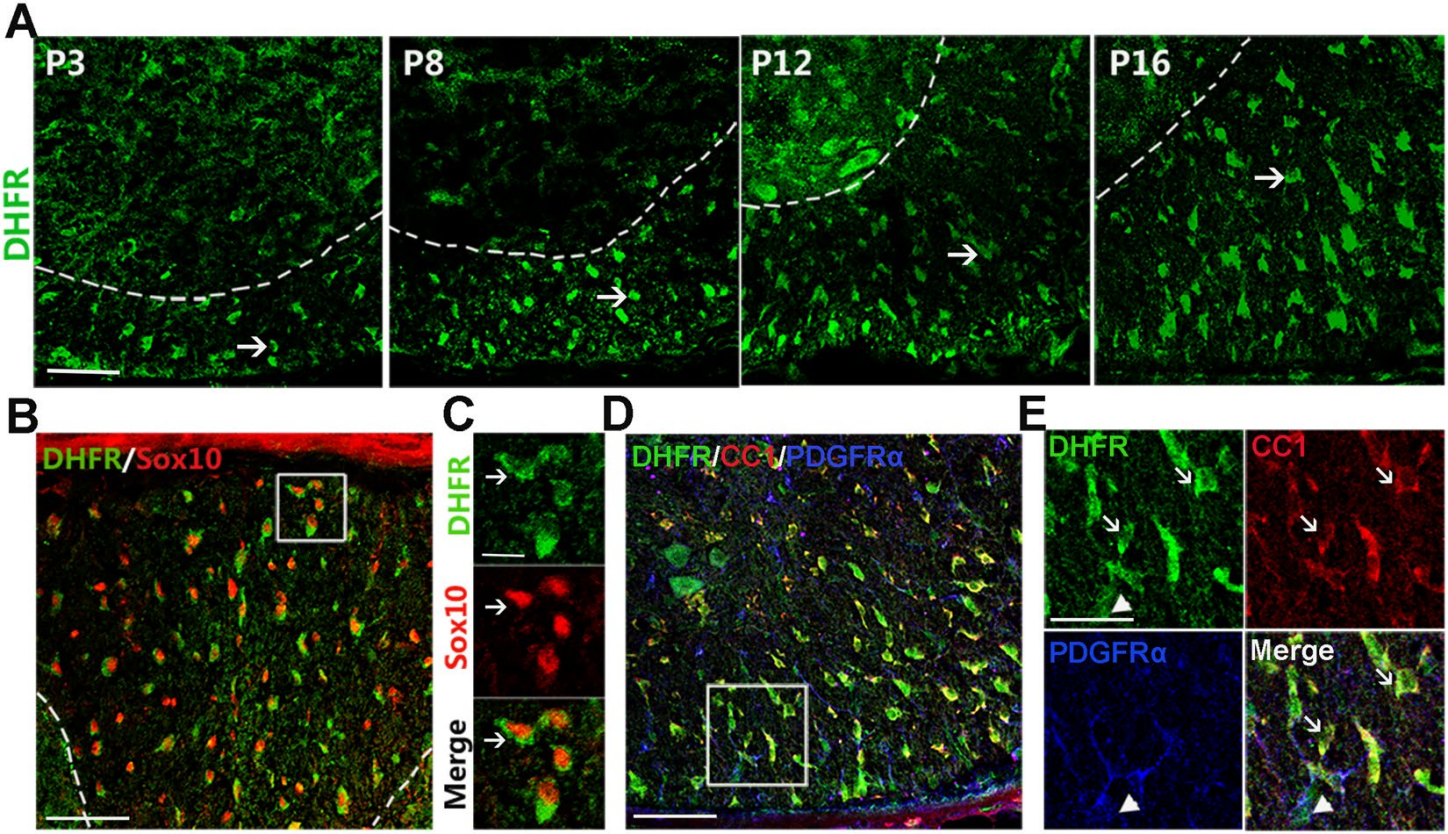
DHFR is highly expressed in oligodendrocytes. (**A**) Spinal cords isolated from the wild type mice immunostained with DHFR antibody at P3, P8, P12 and P16. The ventral parts showed in the images. (**B**,**C**) The spinal cord of wild type mice at P15 is immunostained for DHFR and Sox10. The dorsal part showed in the image. A high magnification of (**B**) is shown in (**C**). Arrows indicate co-labeled cells. (**D**,**E**) The spinal cord of wild type mice at P15 is immunostained for DHFR, CC1, and PDGFRα, respectively. The ventral part showed in the image. A high magnification of (**E**) is shown in (**F**). Arrows indicated DHFR+/CC1+ cells. Arrowhead indicated DHFR+/PDGFRα+ cells. Scale bars: 100 μm (**A**,**B**,**D**), 40 μm (**C**,**E**).

In light of DHFR activation in folate-mediated oligodendrocyte maturation, we first examined if DHFR was expressed in oligodendrocytes. In the developing spinal cord, we detected intense DHFR expression in the white matter region (Fig. 3A). To identify DHFR-expressing cell types, DHFR was co-immunostained with an oligodendrocyte lineage marker, Sox10. DHFR was detected in the majority of Sox10^+^ oligodendrocytes and highly expressed in the cytoplasm (Fig. 3B,C). To further determine the developmental state of DHFR^+^ cells in the oligodendrocyte lineage, we co-labeled DHFR with the markers for differentiated oligodendrocytes (adenomatous polyposis coli protein, CC1^+^) and their precursors (platelet-derived growth factor receptor α, PDGFRα^+^). The results indicated that DHFR was not only strongly expressed in CC1^+^ differentiated oligodendrocytes but also expressed in PDGFRα^+^ OPCs in the spinal white matter (Fig. 3D,E). These observations suggest that DHFR is highly enriched in the oligodendrocyte lineage in the developing CNS.

**Figure 4:**
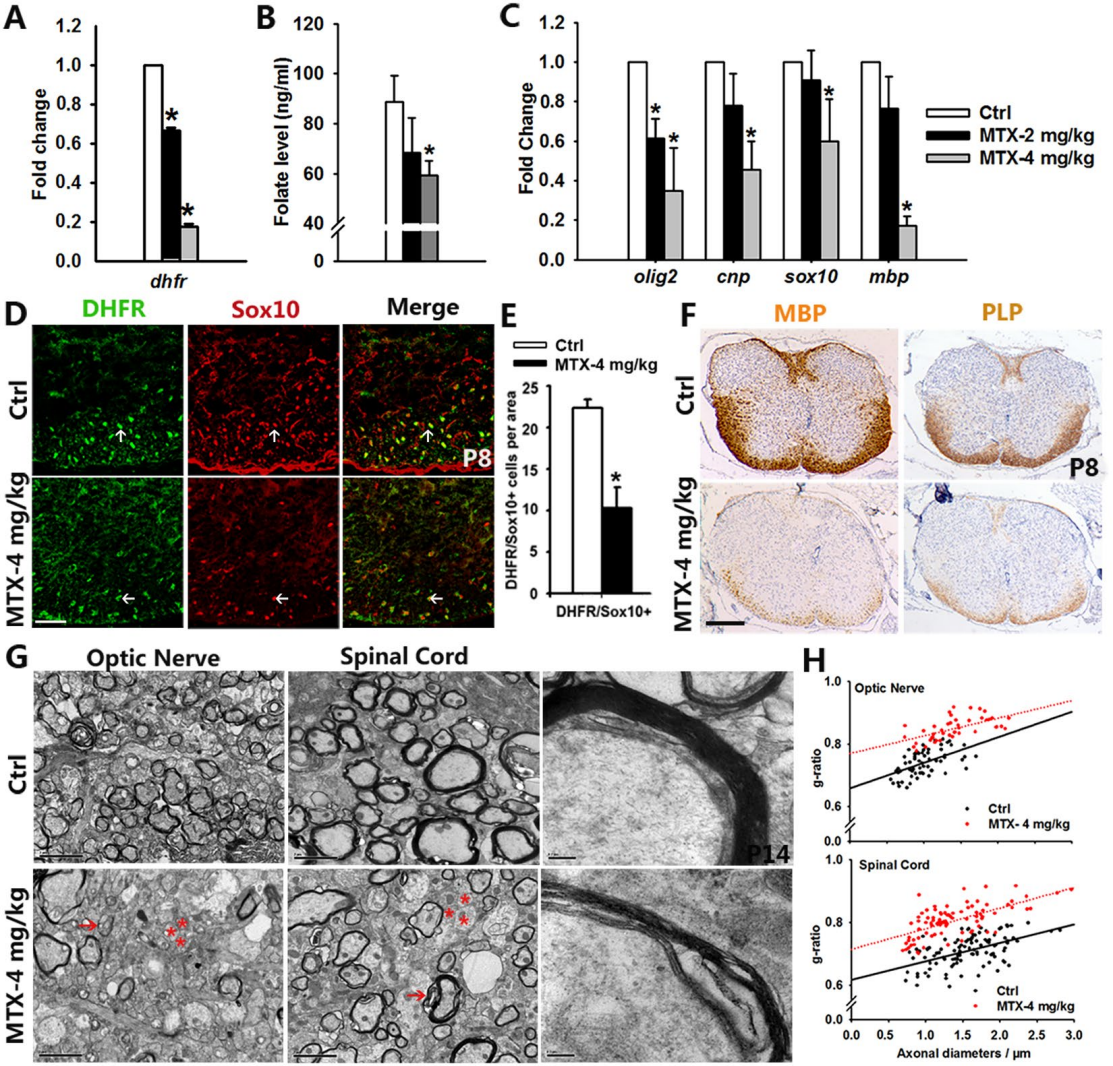
DHFR inhibition triggers evident oligodendrocyte damage and abnormal myelination. (**A**,**C**) qRT-PCR analysis of *Dhfr* mRNA level (**A**) or oligodendrocytes associated genes expression (**C**) in spinal cord from control and different doses of MTX-treated mice at P8. Data represents the mean ± S.D. (n > 3, *p < *0*.*05* compared with control, one-way ANOVA). (**B**) ELISA analysis of folate level in serum isolated from control and MTX-treated mice at P15. Data represents the mean ± S.D. (n = 5, *p < *0*.*05* compared with control, one-way ANOVA). (**D**) The spinal cords isolated from control and MTX-treated mice stained with DHFR and Sox10 antibodies at P8. The ventral parts showed in the images, and arrows indicate co-labeled cells. (**E**) Quantification of the numbers of DHFR+/Sox10+ cells per area (0.16 mm^2^) in (**D**). Data represents the mean ± S.D. (n = 4, *p < *0*.*05* compared with control, student’s t test). (**F**) Immunohistochemistry analysis of spinal cords isolated from control and MTX-treated mice stained with MBP and PLP antibodies at P8. (**G**) Electron micrographs of spinal cord and optic nerve from control and MTX-treated mice at P15. Multilamellar myelin sheaths are compact around axons of control mice, whereas the myelin sheaths around axons from MTX-treated mice are essentially loose. Arrows represent the demyelinated axons, and asterisks indicate the unmyelinated axons. (**H**) G-ratio of individual axons as a function of axonal diameter in spinal cord and optic nerve from control and MTX-treated mice. (n > 100) Scale bars: 100 μm (**D**), 200 μm (**F**), 2 μm ((**G**) left), 0.2 μm ((**G**) right).

DHFR, a key enzyme in folate metabolism, converts dihydrofolate into tetrahydrofolate. To determine the role of DHFR in oligodendrogenesis, we used a pharmacological inhibitor methotrexate (MTX) to inhibit DHFR *in vivo*. Different doses of MTX (2 mg/kg or 4 mg/kg) were intraperitoneally injected into pregnant mice and pups at E8.5, E15.5, P0 or P7, and the spinal cord or brain at different ages of pups were collected and analyzed^36^. MTX injection resulted in a dose-dependent downregulation of the DHFR mRNA level in the spinal cord and the folate level in serum (Fig. 4A,B). In addition, the expression of DHFR in Sox10-expressing cells decreased significantly in spinal cords of DHFR inhibition mice (DHFRi mice) (Fig. 4D,E), suggesting that DHFR expression in oligodendrocytes was reduced after MTX treatment. qPCR analysis indicated a significant downregulation of myelin genes such as *Cnp* and *Mbp*, and the genes encoding crucial differentiation activators such as *Sox10* and *Olig2* in DHFRi mice (Fig. 4C). In addition, expression of MBP and PLP was notably reduced in the spinal cord from DHFRi mice compared to control mice (Fig. 4F). Collectively, these data suggest that DHFR inhibition by MTX causes oligodendrocyte differentiation defects.

To determine whether inhibition of folate/DHFR could cause a permanent defect on myelination, we injected MTX from the embryonic stage E8.5 to P14 weekly (Fig. S2A). MTX was then withdrawn after P15. Despite downregulation after MTX treatment at P14, MBP and PLP expression increased in DHFRi mice at P21 and P28 after MTX withdrawal (Fig. S2B), indicating the oligodendrocyte differentiation process can be re-initiated in the absence of MTX.

### DHFR inhibition results in severe myelination deficiency

In light of our data demonstrating that expression of mature oligodendrocyte markers was reduced in DHFRi mice, we further investigated myelin sheath assembly in the CNS by electron microscopy. In contrast to abundant myelinated axons observed in control mice, the number of myelinated axons was significantly reduced either in spinal cords or in optic nerves of DHFRi mice (Fig. 4G). The few myelinated axons were characterized by thinner myelin sheaths exhibiting a higher g-ratio (the ratio of the inner axonal diameter to the total outer diameter) (Fig. 4G right, H) in both spinal cords and optic nerves of DHFRi mice compared to control mice. Therefore, we conclude that DHFR inhibition severely impairs developmental myelination.

### DHFR inhibition causes oligodendrocyte differentiation defects and death

**Figure 5:**
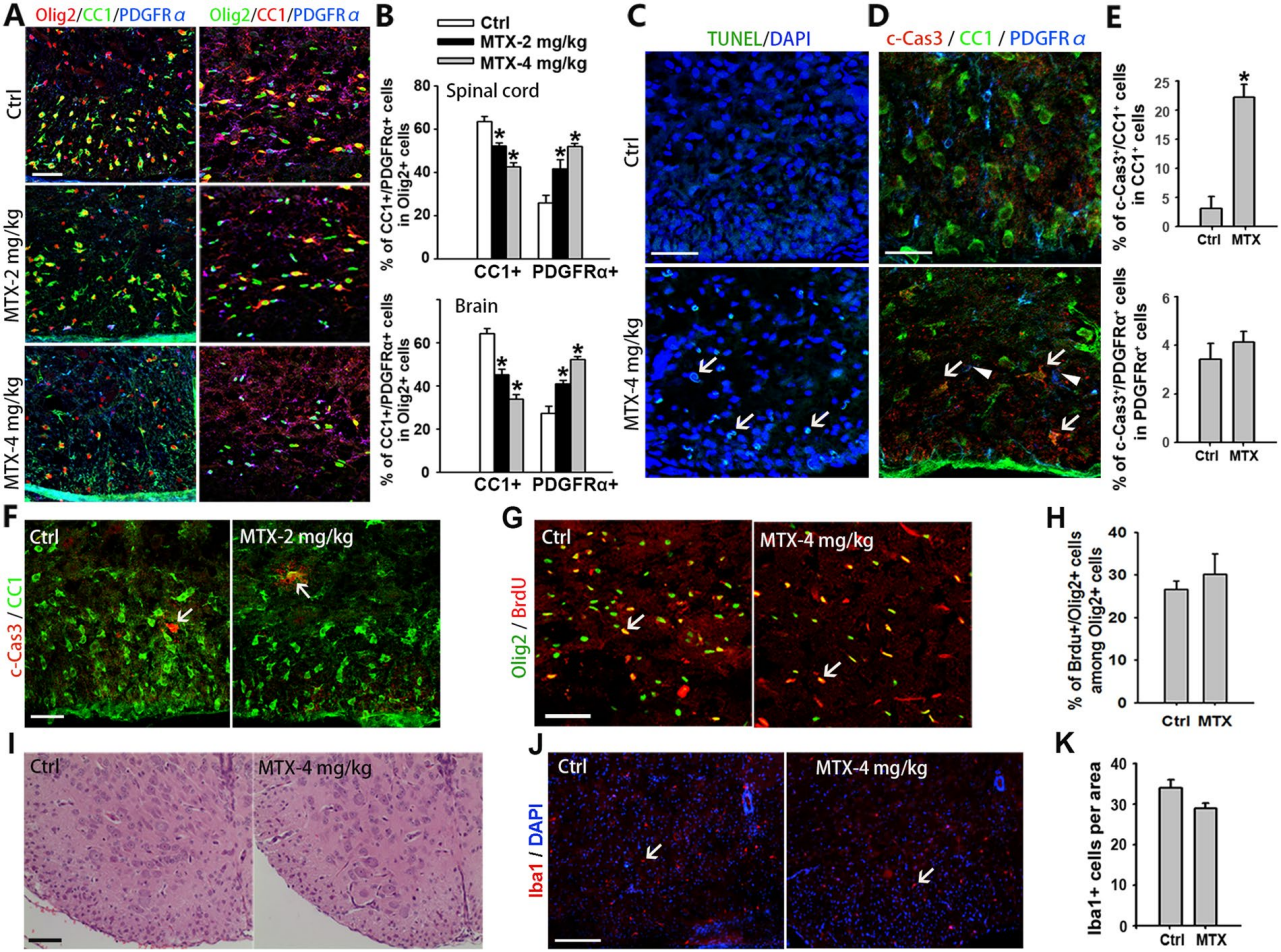
DHFR inhibition causes oligodendrocyte death and differentiation restrain. (**A**) Immunostaining of Olig2, CC1 and PDGFRα antibodies on spinal cord (left) and corpus callosum of brain (right) from control and MTX (2 mg/kg, 4 mg/kg)-treated mice at P8. (**B**) Quantification of the proportion of CC1+ or PDGFRα+ cells among Olig2+ cells in both spinal cord and brain of control and MTX (2 mg/kg, 4 mg/kg)-treated mice at P8 in (**A**). Data represents the mean ± S.D. (n = 5, *p < *0*.*05* compared with control, one-way ANOVA). (**C**) Spinal cord isolated from control and MTX (4 mg/kg)-treated mice at P8 detected oligodendrocyte death by TUNEL assay. The ventral parts showed in the images. Arrows indicate TUNEL-positive cells. (**D**–**F**) Spinal cord isolated from control and MTX (2 mg/kg, 4 mg/kg)-treated mice at P8 detected oligodendrocyte death by co-staining with cleaved-Caspase 3 (c-Cas 3), CC1 and PDGFRα antibodies. The ventral parts showed in the images. Arrows indicate CC1 and cleaved-Caspase 3 double positive cells, arrowheads show PDGFRα+ cells. Quantification of the number of c-Cas 3+ cells among CC1+ or PDGFRα+ cells in (**D**) was shown in (**E**). (**G**) Immunostaining using antibodies to BrdU and Olig2 in cerebral white matter of control and MTX (4 mg/kg)-treated mice at P8. Arrows indicate BrdU+/Olig2+ cells. (**H**) Quantification of the number of BrdU+/Olig2+ cells among Olig2+ cells in cerebral white matter of (**G**). Data represents the mean ± S.D. (n = 5). (**I**) Hematoxylin-eosin (**H**,**E**) staining is used for detecting inflammation in spinal cords from control and MTX (4 mg/kg)-treated mice at P8. (**J**) Immunostaining with Iba1 antibody in spinal cord of control and MTX (4 mg/kg)-treated mice at P8. The ventral parts showed in the images. Arrows indicate the Iba1+ cells. (**K**) Quantification of the numbers of Iba1+ cells per area (0.1 mm^2^) in (**J**). Data represents the mean ± S.D. (n = 5). Scale bars: 25 μm (**D**), 50 μm (**A**,**C**,**F**,**G**), 100 μm (**I**,**J**).

To examine the fate of oligodendrocytes after DHFR inhibition, we carried out immunostaining of Olig2, CC1 and PDGFRα in the CNS. DHFR inhibition resulted in oligodendrocyte differentiation defects (Fig. 5A). The proportion of CC1^+^ cells among Olig2^+^ cells in spinal cords from DHFRi mice at P8 significantly decreased compared with control mice. Conversely, the proportion of PDGFRα and Olig2 double positive cells increased with MTX treatment. Similar results were observed in the cerebral white matter from control and DHFRi mice (Fig. 5A,B). Consistent with this, western blotting analysis showed that MBP expression decreased in the spinal cord of DHFRi mice at P15, while expression of the OPC marker PDGFRα increased (Fig. S3). To determine whether abnormal development of oligodendrocytes could be caused by cell death in DHFRi mice, we examined the expression of TUNEL and active cleaved-Caspase 3 (c-Cas3) in spinal cords of DHFRi mice. In contrast to control mice, substantial cell death was detected in the spinal white matter of DHFRi mice by TUNEL assay (Fig. 5C). To further identify the dying cell types, c-Cas3, CC1 and PDGFRα were co-immunostained in the spinal cord from control and DHFRi mice. We detected the number of CC1 and c-Cas3 double positive cells increased in the spinal cord of DHFRi mice (Fig. 5D,E), indicating that DHFR inhibition leads to oligodendrocyte death. We detected that few PDGFRα+ cells were co-stained with c-Cas3 in DHFRi mice (Fig. 5D,E), suggesting that DHFR inhibition caused the death of mature oligodendrocytes rather than OPCs. Furthermore, no obvious oligodendrocyte death was observed in DHFRi mice with low doses of MTX (2 mg/kg) (Fig. 5F), suggesting a direct effect of low folate/DHFR levels on oligodendrocyte differentiation. In addition, BrdU pulse-labeling experiments indicated that the proliferative rate of OPCs in the cerebral white matter of DHFRi mice was comparable to control mice (Fig. 5G,H). Taken together, DHFR inhibition blocks oligodendrocyte differentiation and induces mature oligodendrocyte death.

To determine whether inflammation may contribute to oligodendrocyte defects in DHFRi mice, hematoxylin-eosin (HE) staining was carried out in spinal white matter. We did not detect substantial inflammatory cell infiltration in the developing spinal cord of DHFRi mice at P8 (Fig. 5I). In addition, no significant activation of microglia was found in DHFRi mice by Iba1 staining (Fig. 5J,K). Collectively, these data indicate that DHFR inhibition causes oligodendrocyte differentiation defects and mature oligodendrocyte death, while keeping oligodendroglial lineage cells at the precursor stage.

### Folate partially rescues oligodendrocyte defects caused by DHFR inhibition

**Figure 6:**
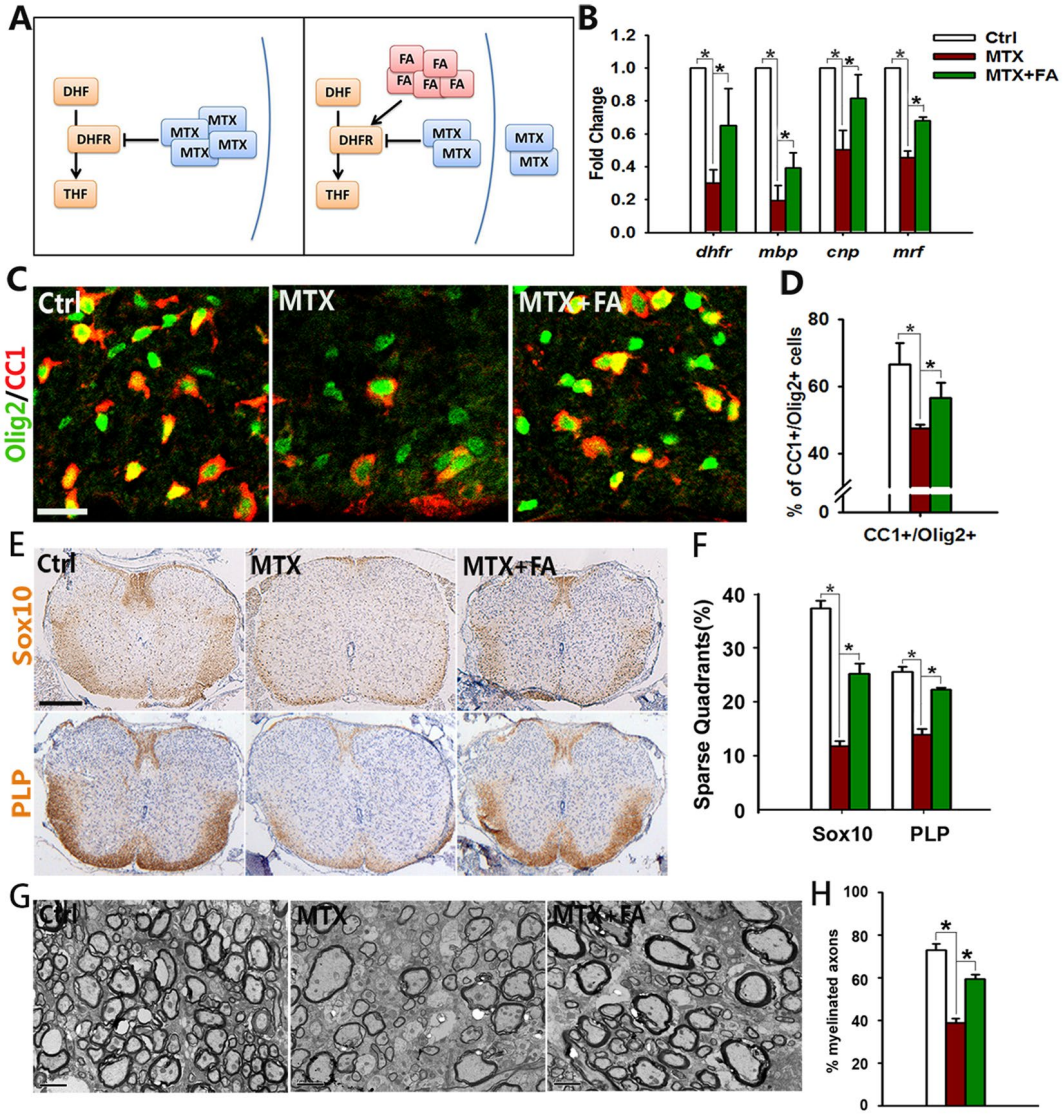
Oligodendrocyte defects induced by DHFR inhibition could be rescued by folate supplement. (**A**) Diagram shows the competition of folate and MTX on DHFR. Folate can block MTX entering cells by competitive combination of membrane receptor, leading to the reduction of DHFR inhibition. (**B**) qRT-PCR is carried out to analyze mRNA level of *Dhfr*, *Mbp*, *Cnp*, *Myrf* of spinal cords form control, MTX (4 mg/kg) and MTX (4 mg/kg) plus folate (10 mg/kg) mice at P8. Data represents the mean ± S.D. (n > 3, *p < *0*.*05*, one-way ANOVA). (**C**) Immunostaining using antibodies of Olig2, CC1 on spinal cord from control, MTX (4 mg/kg) and MTX (4 mg/kg) plus folate (10 mg/kg) mice at P8. The ventral parts showed in the images. (**D**) Quantification of the percentage of CC1+ cells among Olig2+ cells in (**C**). Data represents the mean ± S.D. (n = 5, *p < *0*.*05*, one-way ANOVA). (**E**) Immunohistochemistry using antibodies of Sox10 and PLP on spinal cord from control, MTX (4 mg/kg) and MTX (4 mg/kg) plus folate (10 mg/kg) mice at P8. (**F**) The number of quadrants with sparse staining for Sox10 and PLP in (**E**) scored and expressed as a percentage of the total number of quadrants. (n = 5, *p < *0*.*05*, one-way ANOVA). (**G**,**H**) Electron micrographs of spinal cord from control, MTX (4 mg/kg) and MTX (4 mg/kg) plus folate (10 mg/kg) mice at P15. (**H**) Shows the percentage of myelinated axons. Data represent the mean ± S.E.M from three animals (*p < *0*.*05*, one-way ANOVA). Scale bars: 50 μm (**C**), 200 μm (**E**), 2 μm (**G**).

Given the essential role of DHFR in oligodendrocyte maturation and that folate could effectively compete with MTX for DHFR activity (Fig. 6A), we then tested whether folate was able to rescue the oligodendrocyte defects caused by DHFR inhibition. Folate (10 mg/kg) was administrated intragastrically daily during the period of MTX treatment from E8.5 to P7 weekly, and pups were sacrificed at P8. qRT-PCR results showed that folate treatment increased the expression of *Dhfr* in the spinal cord at P8 (Fig. 6B, left bar). Similarly, an increase of myelin-associated genes such as *Mbp*, *Cnp* and *Myrf* was observed in folate-treated mice (Fig. 6B). Folate supplement substantially increased CC1+/Olig2+ mature oligodendrocytes (Fig. 6C,D). Similarly, Sox10 and PLP immunoreactivity was upregulated following folate treatment (Fig. 6E,F). Furthermore, electron microscopy analysis showed that folate supplement increased the percentage of myelinated axons at P15. (Fig. 6G,H). Taken together, these results indicate that folate, at least partially, is able to rescue the oligodendrocyte defects caused by MTX-inhibited DHFR activity.

### Folate/DHFR regulates oligodendrocyte development by activating AMPKα phosphorylation

**Figure 7:**
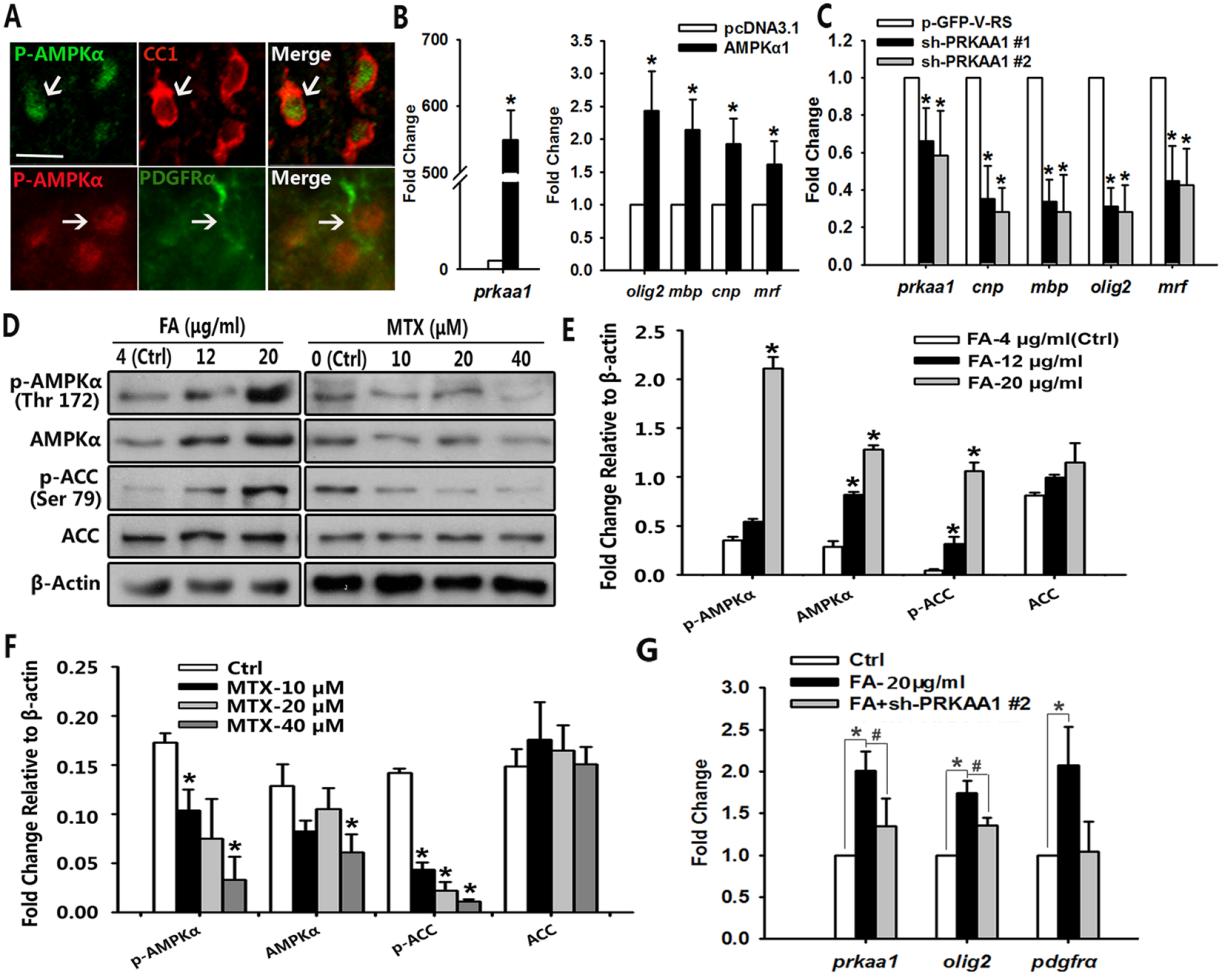
AMPK activity is involved in DHFR regulation of oligodendrocyte development. (**A**) The spinal cord of wild type mice at P8 was co-labeled for p-AMPKα with PDGFRα and CC1. Arrows indicate co-labeled cells. (**B**) qRT-PCR analysis of *Prkaa1*, *Olig2*, *Mbp*, *Cnp* and *Myrf* genes expression in Oli-neu cells transfected with AMPKα1 and control plasmids for 48 h. Data represents the mean ± S.D. (n > 3,*p < *0*.*05* compared with the control, student’s t test). (**C**) qRT-PCR analysis of *Prkaa1*, *Olig2*, *Mbp*, *Cnp* and *Myrf* genes expression in Oli-neu cells transfected with AMPKα1 sh-RNA (PRKAA1 #1 and #2) and control shRNA for 48 h. Data represents the mean ± S.D. (n > 3,*p < *0*.*05* compared with the control, one-way ANOVA). (**D**) Western blot analysis of p-AMPKα (60 kDa), AMPKα (60 kDa), p-ACC (280 kDa) and ACC (280 kDa) proteins expression in Oli-neu cells with folate or MTX treatment for 48 h compared to the individual control. β-actin (43 kDa) is the loading control. (**E**,**F**) Expression level of p-AMPKα, AMPKα, p-ACC and ACC proteins expression relative to β-actin of (**D**). Data represents the mean ± S.D. (n > 3, *p < *0*.*05* compared with the control, one-way ANOVA). (**G**) qRT-PCR analysis of *Prkaa1*, *Olig2* and *Pdgfrα* genes expression in Oli-neu cells treated with folate (20 μg/ml) in the absence or presence of AMPKα shRNA#2 for 48 h. Data represents the mean ± S.D. (n > 3, *p < *0*.*05*, one-way ANOVA). Scale bars: 40 μm (**A**).

Folate is essential for one-carbon groups transfer, which is closely associated with cellular energy metabolism, characterized by the phosphorylated activation of AMP-activated protein kinase (AMPK)^23^. Based on this, we first confirmed p-AMPKα expression in PDGFRα^+^ and CC1^+^ oligodendrocytes (Fig. 7A). To determine the potential role of AMPKα in oligodendrocyte differentiation, we transfected Oli-neu cells with expression vectors carrying a control or AMPKα cDNA or shRNA to test whether AMPKα levels affected oligodendrocyte differentiation. Overexpression of AMPKα1, confirmed by qPCR (AMPKα1 was encoded by *Prkaa1* gene, Fig. 7B left), led to a significant increase of *Olig2*, *Mbp*, *Cnp* and *Myrf* mRNA levels (Fig. 7B right). Conversely, expression of these genes was reduced with AMPKα1 silencing (Fig. 7C). Thus, these gain or loss of function data indicate that AMPKα promotes oligodendrocyte differentiation *in vitro*.

To investigate whether AMPK mediates the folate/DHFR function in oligodendrocyte development, we examined the level of p-AMPKα following the treatment of folate and MTX *in vitro*, respectively. Western blotting analysis showed that expression of p-AMPKα and AMPKα increased after folate treatment in Oli-neu cells. Folate treatment activated the phosphorylation of AMPK downstream target, acetyl CoA carboxylase (ACC, Fig. 7D left, E). Conversely, expression of p-AMPKα, AMPKα and p-ACC proteins notably decreased after DHFR inhibitor treatment in a concentration-dependent way (Fig. 7D right, F). Consistently, folate supplement increased *Prkaa1* gene expression significantly assayed by qRT-PCR (Fig. 7G left bar). Furthermore, *Olig2* and *Pdgfrα* expression was significantly decreased by *Prkaa1* knockdown compared with folate treatment alone (Fig. 7G). This suggests that AMPKα1 silencing abolishes folate-induced oligodendrocyte differentiation in Oli-neu cells. Taken together, these data suggest that AMPKα activation is, at least partially, involved in oligodendrocyte differentiation promoted by active folate metabolism.

**Figure 8:**
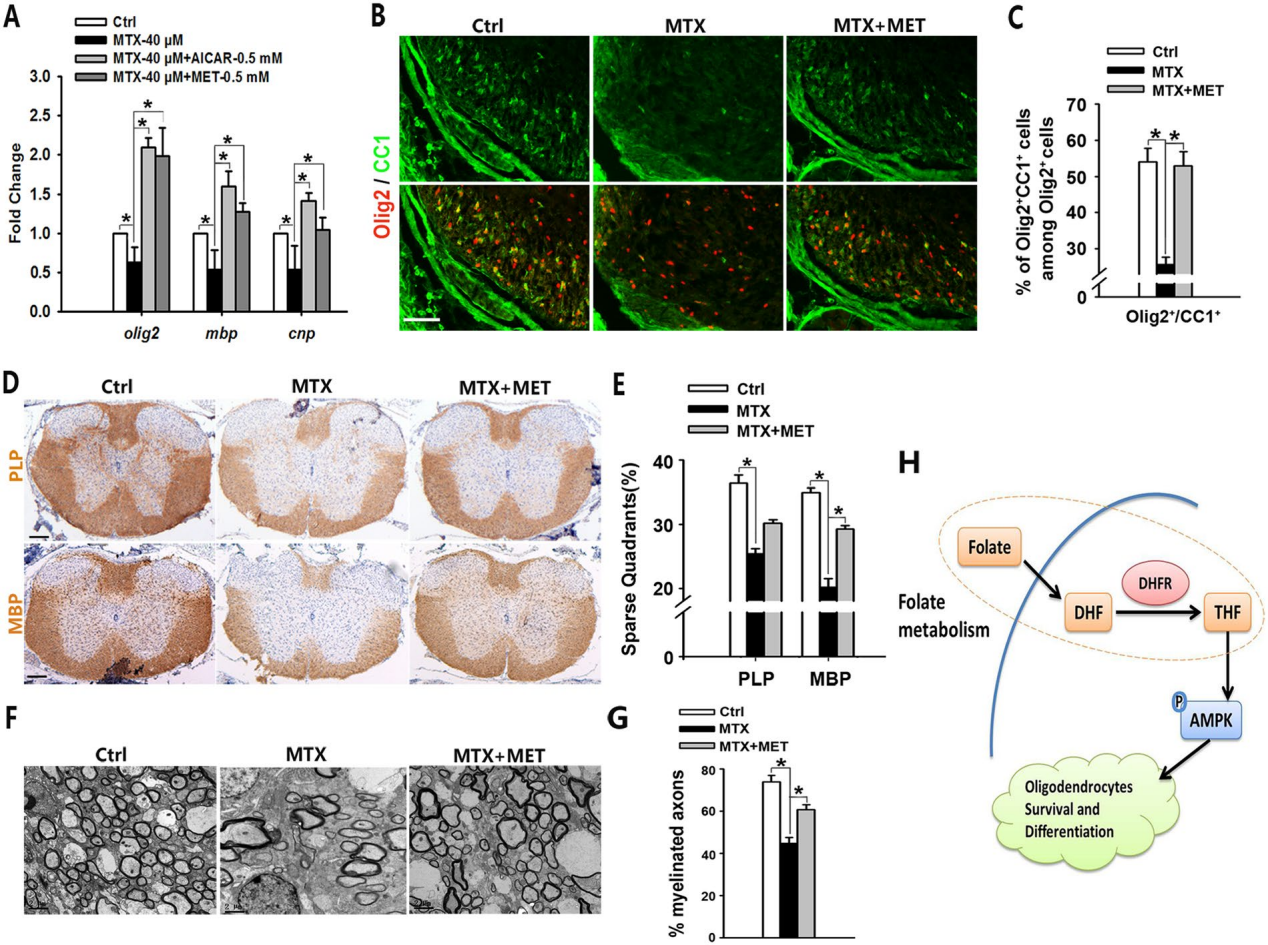
AMPKα activation rescues oligodendrocyte defects caused by DHFR inhibition. (**A**) qRT-PCR is performed to investigate myelin-associated gene expression in Oli-neu cells treated with MTX (40 μM) in the presence of canonical AMPK activators (AICAR or MET) for 48 h. Data represents the mean ± S.D. (n > 3, *p < *0*.*05*, one way ANOVA). (**B**) Immunostaining using antibodies of Olig2, CC1 on spinal cord from control, MTX (4 mg/kg) and MTX (4 mg/kg) plus MET (100 mg/kg) mice at P8. The ventral parts showed in the images. (**C**) Quantification of the percentage of CC1+ cells among Olig2+ cells in (**B**). Data represents the mean ± S.D. (n = 5, *p < *0*.*05*, one way ANOVA). (**D**) Immunostaining of the spinal cords isolated from control, MTX (4 mg/kg) and MTX (4 mg/kg) plus MET (100 mg/kg) treated mice at P15 stained with PLP and MBP antibodies. (**E**) The number of quadrants with sparse staining for PLP and MBP of (**D**) was scored and expressed as a percentage of the total number of quadrants. (n = 5, *p < *0*.*05*, one way ANOVA). (**F**,**G**) Electron micrographs of spinal cord from control, MTX (4 mg/kg) and MTX (4 mg/kg) plus MET (100 mg/kg) mice at P15. (**H**) Shows the percentage of myelinated axons. Data represent the mean ± S.E.M. from three animals (*p < *0*.*05*, one-way ANOVA). (**H**) Diagram illustrates the regulation of folate/DHFR on oligodendrocyte maturation. Folate is able to promote oligodendrocyte maturation both *in vitro* and *in vivo*. On the contrary, folate/DHFR inhibition causes severe oligodendrocyte injury and myelin defects. AMPKα, a potential folate/DHFR-activated target, plays a key role on folate/DHFR regulation of oligodendrocyte maturation. Together, folate/DHFR/AMPKα axis is an important regulator to promote oligodendrocyte survival and differentiation. Scale bars: 100 μm (**B**,**D**), 2 μm (**F**).

To investigate if AMPKα activator could rescue oligodendrocyte defects caused by abnormal folate metabolism. Treatment of canonical AMPKα activators, either AICAR or metformin (MET), could reverse MTX-induced reduction of *Olig2*, *Mbp* and *Cnp* genes expression in Oli-neu cells (Fig. 8A). This suggests that activation of AMPKα could antagonize oligodendrocyte defects caused by DHFR inhibition. We further investigated whether metformin-activated AMPKα could protect oligodendrocyte defects caused by DHFR inhibition *in vivo*. MTX was administered into pregnant mice or pups at E8.5, P0, P7, P14 via intraperitoneal injection, and metformin was intraperitoneally injected into pups daily. The percentage of CC1^+^Olig2^+^ mature oligodendrocytes was upregulated after MET administration compared with DHFRi mice (Fig. 8B,C). Similarly, in contrast to MTX-injured spinal cord at P15 (Fig. 8D middle), metformin treatment partially antagonized MTX-induced oligodendrocyte defects via restoring MBP and PLP expression in the spinal cord (Fig. 8D right, E). Furthermore, electron microscopy showed that metformin enhances the percentage of myelinated axons from MTX-induced injury (Fig. 8F,G). Collectively, our data suggest that AMPKα activation ameliorates DHFR inhibition-induced oligodendrocyte defects.

## Discussion

Folate is known to play an important role in CNS development and disorders mainly via the promotion of neuron differentiation and axon regrowth^37–40^. Here we show a novel role of folate metabolism in oligodendrocyte myelination (Fig. 8H). Our data indicate that folate is sufficient to promote oligodendrocyte maturation and survival. We find that oligodendrocyte-expressing DHFR, a key enzyme in folate metabolism, is required for oligodendrocyte survival and myelination through regulating a downstream effector AMPKα. Together, our findings point to a folate/DHFR/AMPKα axis as an important regulator in promoting oligodendrocyte maturation and survival.

It has been reported that chronic folate depletion causes severe neural tube defects (NTDs)^41, 42^, characterized by massive neuron loss and thickness decrease in the hippocampal CA1 pyramidal layer^33, 43^. In this study, we used a modified version of a previously established folate-deficient mouse model to investigate its role on oligodendrocyte development with administration of low folate-supplemented diet prior to pregnancy. Our results showed that a low folate-diet intake caused oligodendrocyte differentiation defects without neuronal loss, suggesting the oligodendrocyte defects occurred prior to neuronal loss when folate is deficient.

Although treatment of MTX, a DHFR activity inhibitor, has been shown to induce upregulation of DHFR gene expression^44^, maybe due to a feedback mechanism, it may also cause decreased DHFR mRNA expression in other contexts^45^. We found that DHFR mRNA and protein levels were downregulated in the spinal cord after MTX treatment. This is likely attributed to the decrease of oligodendrocytes, which express high mRNA levels of the *DHFR* gene.

Recent studies indicate that presenilin-1 mutation increases the vulnerability of oligodendrocytes to folate deficiency *in vitro* ^46^, and that folate deficiency has no effects on MBP expression in the maternal brain and spinal cord^47^. Here our present study provides evidence that folate deficiency by low dietary folate during pregnancy and lactation causes defects in oligodendrocyte development and myelination. Folate deficiency inhibits OPC differentiation. In addition, folate reduction by pharmacological DHFR inhibition results in oligodendrocyte death and differentiation defects. Taken together, myelination defects caused by folate deficiency are attributed to a direct effect on OPC maturation and an indirect effect on mature oligodendroglial survival. Folate deficiency has been associated with myelin lipid abnormalities and impaired lipid metabolism^16^. It is possible that that altered myelin lipid metabolism due to folate deficiency might result in myelination defects.

The mechanisms underlying folate/DHFR regulation of oligodendrocyte myelination are not fully understood. It has been reported that folate metabolism is related to DNA synthesis and methylation. Patients with DHFR mutations present no hyperhomocysteinaemia, indicating that the neurological defect caused by DHFR deficiency does not include the methylation cycle but is probably in the DNA-synthesis arm of folate metabolism^8^. DHFR converts dihydrofolate into tetrahydrofolate, which is required for AMP synthesis and AMPK activity. Consistently, our results show that expression of p-AMPKα and AMPKα is enhanced with folate supplement, while downregulated by DHFR inhibition. These data indicate that AMPKα plays a critical role in folate/DHFR-regulated oligodendrocyte maturation. At present, however, how DHFR regulates AMPKα activity remains to be determined. Nonetheless, we show that AMPKα activation can reverse the oligodendrocyte differentiation defect caused by DHFR inhibition, suggesting that a folate/DHFR/AMPKα regulatory axis is crucial for oligodendrocyte survival and differentiation.

## Materials and Methods

### Animals

Adult C57BL/6 mice (8–10 w) were obtained from SHANGHAI SLAC LABORATORY ANIMAL CO. LTD. Adult C57BL/6 mice were crossed with each other to generate embryos and pups.

Statements of Ethical Approval: The animal studies have been approved by the Institutional Animal Care & Use Committee (IACUC) at Zhejiang University, with ethical approval number IACUC-15012, and all experimental protocols were conducted in accordance with institutional guidelines.

### Compound intervention

For OPCs and Oli-neu cells, folate was dissolved in 0.4% NaOH solution, methotrexate (canonical DHFR inhibitor) was dissolved in 0.9% NaCl solution at proper concentration. For folate supplement and deficiency, pregnant mice were fed different folate-supplemented diet. For DHFR inhibition, mice were intraperitoneally injected with methotrexate (2 or 4 mg/kg weight) on indicated day. For AMPKα activation, metformin (100 mg/kg) was intragastricly or intraperitoneally administrated to pregnant and postnatal mice. Meanwhile, control mice took in equal amounts of vehicle (0.9% NaCl solution).

### Primary culture of Oligodendroglia Progenitor Cell (OPC)

Isolation and culture protocol of rat cortical oligodendrocyte precursors from pups at P2 was described earlier^48^. OPCs should be planted on poly-lysine coated coverslips and kept in SATO medium supplemented with proliferation (10 ng/ml PDGF-AA) growth factors. In this study, DMEM with no folic acid (Sigma, D2429) was used for OPCs cultures, and indicated concentrations of folate were added manually. The extent of oligodendrocyte process outgrowth was measured by the area surrounding the nuclei including the outermost tips occupied by processes using Image J analysis software.

### *In situ* hybridization

Nonradioactive RNA *in situ* hybridization was performed as previously described^49^. *Mbp* and *Plp1* gene probes were used in the experiment. Detailed protocols are available upon request.

### Immunofluorescence and immunohistochemistry

Tissues were fixed with 4% paraformaldehyde (PFA) and embedded in O.C.T. compound (Tissue-Tek) for cryo-sections. Brain and spinal cord sections (12–16 μm) were incubated with blocking buffer (PBS with 5% NGS and 0.3% Triton-100) for 1 h. For immunohistochemistry (IHC), 3% H_2_O_2_ needed to be performed to block endogenous peroxidase. Then primary antibodies were incubated at 4 °C overnight. The primary antibodies were as follows: DHFR (Abcam ab85056, 1:500), Olig2 (Millipore Ab9610, 1:5000), CC1 (Calbiochem OP80, 1:50), MBP (Covance SMI-94R, 1:5000), PDGFRα (BD Bioscience 558774, 1:500), Sox10 (R&D systems NL2864R, 1:200), PLP (Millipore Mab388, 1:2000), NeuN (Millipore Mab377, 1:1000), cleaved-Caspase 3 (Cell signal 9661L, 1:1000), BrdU (BD pharmingen 555627, 1:200). Appropriate fluorophore-conjugated secondary antibodies (cy2, cy3 and cy5, Jackson ImmunoResearch) or alternatively the chromogen DAB were used for detection according to manufacturer’s instructions. In particular, sections were deparaffinized using a standard protocol and stained with hematoxylin and eosin as previously described. Cell death was detected using the one step TUNEL apoptosis assay kit (C1088, Beyotime) according to their protocols. Images were acquired with a Zeiss LSM510 Meta fluorescence confocal microscope or a Leica DM2500 microscope.

### RNA extraction and RT-PCR

Total RNA was extracted and purified from tissues and cells using Trizol reagent (Invitrogen) according to manufacturer’s procedures. cDNA synthesis was performed by using TransScript kit (TransGen Biotech). Quantitative RT-PCR was carried out using the ABI Fast 7500 Real-time PCR instrument (Perkin-Elmer Applied Biosystems), and the relative mRNA levels were normalized to internal control such as GAPDH. The PCR primer sequences are available upon request.

### Electron microscopy and g-ratio analysis

The optic nerves and spinal cords of mice were dissected and fixed in fresh fixative overnight at 4 °C. Tissues were rinsed in PBS, postfixed in 1% OsO_4_ in PBS for 1 h, dehydrated in a graded ethanol series, infiltrated with propylene oxide, and embedded in Epon. To calculate g-ratios, measurements were made on electron micrographs from each group. The ratios of the axonal diameter to the myelinated fiber diameter were measured for at least 100 times each group using Image J.

### ELISA and Western blotting

The level of folate in serum was measured by FA/VB9 Elisa kit (E-EL-0009c, Elabscience) according to manufacturer’s instructions. For western blotting, Tissues or cells were collected and lysed in universal lysis/immunoprecipitation buffer (50 mM Tris-HCl, 150 mM NaCl, 2 mM EDTA, 2 mM EGTA, 25 mM NaFl, 25 mM β-glycerophosphate pH 7.5, 0.1 mM sodium orthovanadate, 0.1 mM PMSF, 5 μg of leupeptin per ml, 0.2% Triton X-100, 0.5% Nonidet P-40). The concentrations of the total lysate protein were measured by a standard Bradford assay (Bio-Rad, San Diego, CA). The protein was electrophoresed by S.D.S-PAGE and transferred to nitrocellulose membrane (PierceChemical) and probed with primary antibodies. The primary antibodies were as follows: p-AMPKα (cell signal 2535L, 1:1000), p-ACC (cell signal 3661L, 1:1000), AMPKα (cell signal 5832, 1:1000), ACC (cell signal 3662L, 1:1000), β-Actin (Santa Cruz sc-1615, 1:1000), MBP (Covance SMI-94R, 1:1000), PDGFRα (Santa Cruz sc-338, 1:500). Appropriate secondary antibodies and ECL were performed to visualize the protein signaling.

### Cell culture and Transfection

The oligodendrocyte precursor cell line Oli-neu was graciously provided by Prof. Q. Richard Lu. It was cultured on poly-d-lysine-coated dishes with DMEM supplied with 5% FBS, 2% B27, 1% N2 and 1% horse serum. DHFR, AMPKα1 plasmids (addgene) and shRNA (Origene) were transfected into cells by Lipofectamine 2000 (Invitrogen) according to manufacturer’s procedures. The target sequences of shRNA of AMPKa were as follows: shRNA#1(ATTATGCCGCACCAGAAGTCATTTCAGGA), shRNA#2(CGCAGACTCAGTTCCTGGAGAAAGATGGC).

### Statistical analysis

Quantitative image analysis of Immunohistochemistry and Western blot was performed using Image J analysis software. Especially, for quantification of sparse quadrants, Image J was used to subtract background through setting a threshold to maximum projection grayscale images. The total area of objects and the average size of each object were quantified. The total area occupied by aimed objects was represented as normalized to the first time frame. Quantifications were carried out from at least three independent experiments; data was calculated as mean ± S.D. in the graphs. Results were analyzed using two-tail student’s t test or one way ANOVA, statistical significance was accepted when *p < *0*.*05*.

## Electronic supplementary material

Supplementary data
