# Supplementary material for: Folate Metabolism Regulates Oligodendrocyte Survival and Differentiation by Modulating AMPKα Activity
Source: Sci Rep. 2017 May 11;7:1705. doi: 10.1038/s41598-017-01732-1 (PMC5431811; doi:10.1038/s41598-017-01732-1)

**Folate Metabolism Regulates Oligodendrocyte Survival and Differentiation by Modulating AMPKα Activity**

Qinjie Weng^1,2, ζ^, Jiajia Wang^1, ζ^, Jiaying Wang^1^, Biqin Tan^1^, Jing Wang^1^, Haibo Wang^3^, Tao Zheng^4^, Q. Richard Lu^3^, Bo Yang^1*^, Qiaojun He^1, 2,*^

1. Institute of Pharmacology & Toxicology, Zhejiang Province Key Laboratory of Anti-Cancer Drug Research, College of Pharmaceutical Sciences, Zhejiang University, Hangzhou, China.
2. Center for drug safety Evaluation and Research, Zhejiang University, Hangzhou, China.
3. Department of Pediatrics, Brain Tumor Center, Cancer and Blood Disease Institute, Cincinnati Children’s Hospital Medical Center, OH, USA
4. School of Preclinical and Forensic Medicine, West China Second Hospital, Sichuan University, Chengdu, China.

^ζ^: These authors contributed equally to this work

**Correspondence:**

Dr. Bo Yang

Email: yang924@zju.edu.cn

or

Dr. Qiaojun He

Email: qiaojunhe@zju.edu.cn

Tel/Fax: 86-571-88208400


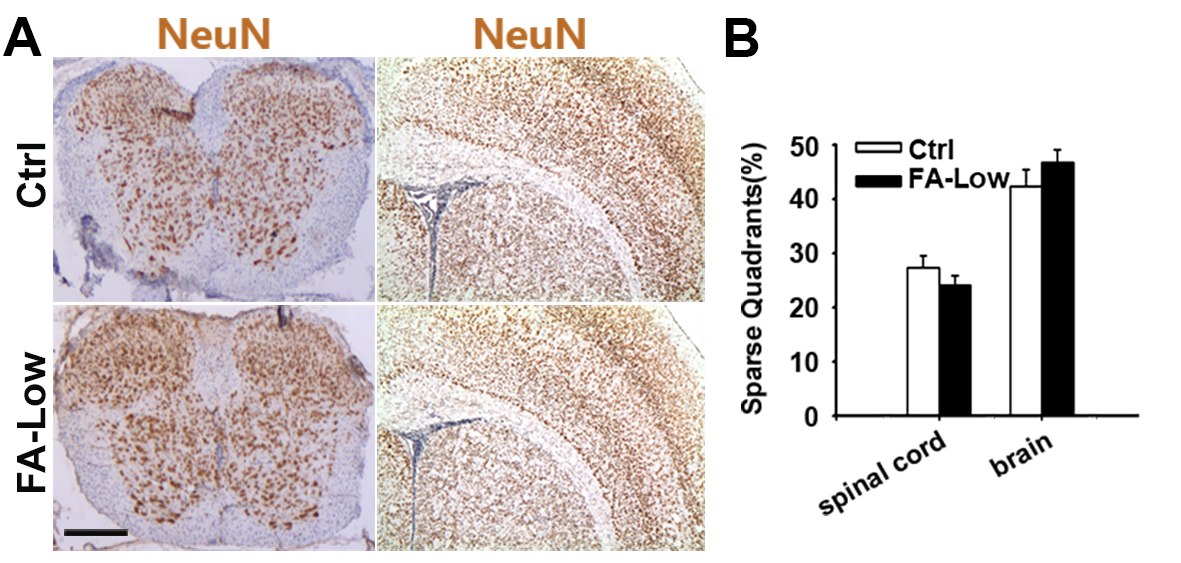


**Figure S1 Low folate intake has no effect on neurons.**
(A) Immunohistochemistry analysis of spinal cords or brains isolated from normal folate-supplemented diet (2 mg folate/kg diet, Ctrl) or low folate-supplemented diet (0.2 mg folate/kg diet, FA-Low) mice stained with NeuN antibody at P8.

(B) The number of quadrants with staining for NeuN of (A) scored and expressed as a percentage of the total number of quadrants. (n=5).
Scale bars: 200 μm (A).


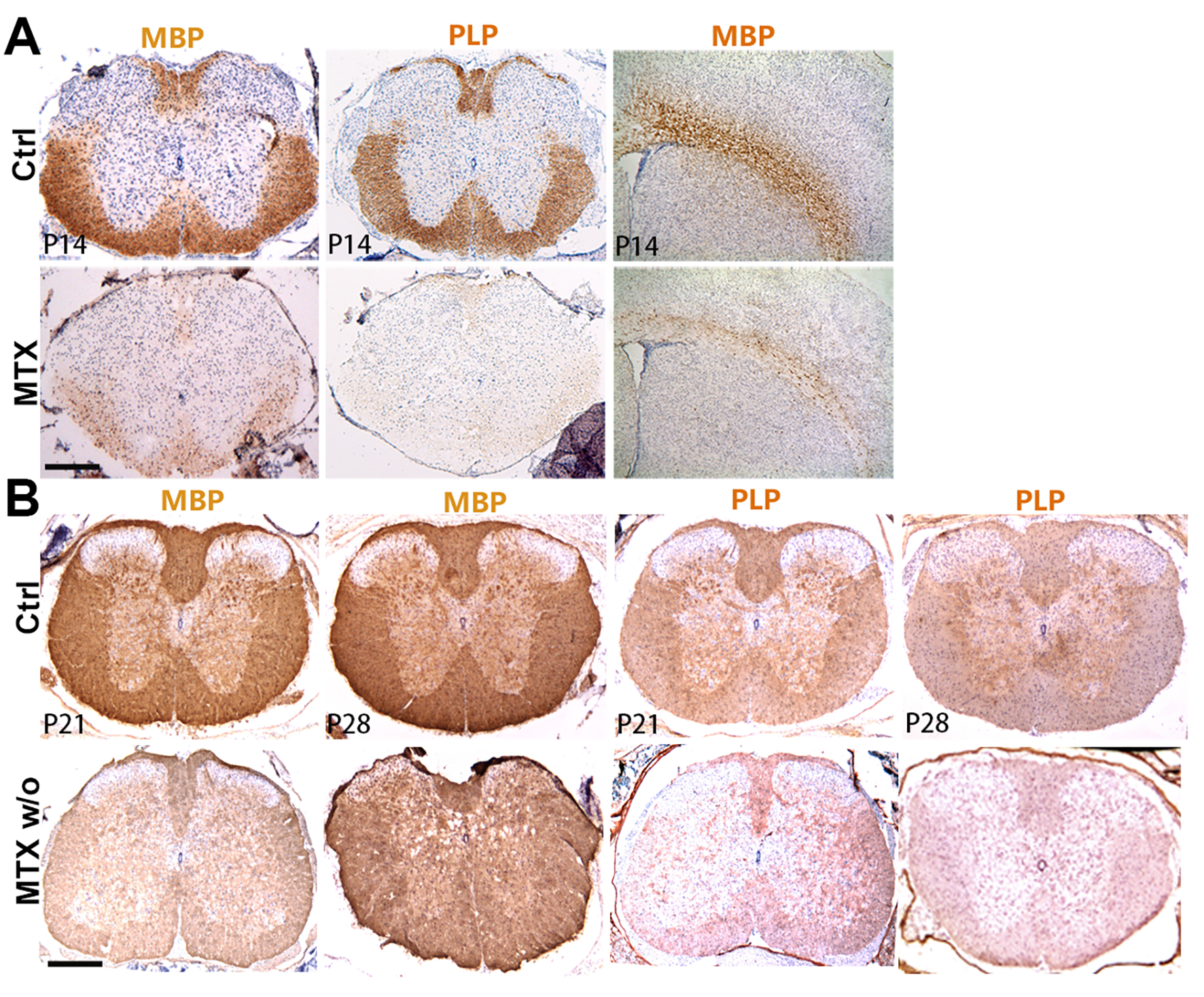


**Figure S2 Oligodendrocyte defects can be recovered when MTX is withdrawn**

(A) Immunostaining of the spinal cord and brain isolated from control, MTX (4 mg/kg) treated mice at P14 stained with PLP and MBP antibodies.

(B) Immunostaining of the spinal cord isolated from control, MTX (4 mg/kg) treated mice at P21 and P28 stained with PLP and MBP antibodies when MTX administration was withdrawn at P15.

Scale bars: 200 μm (A, B).


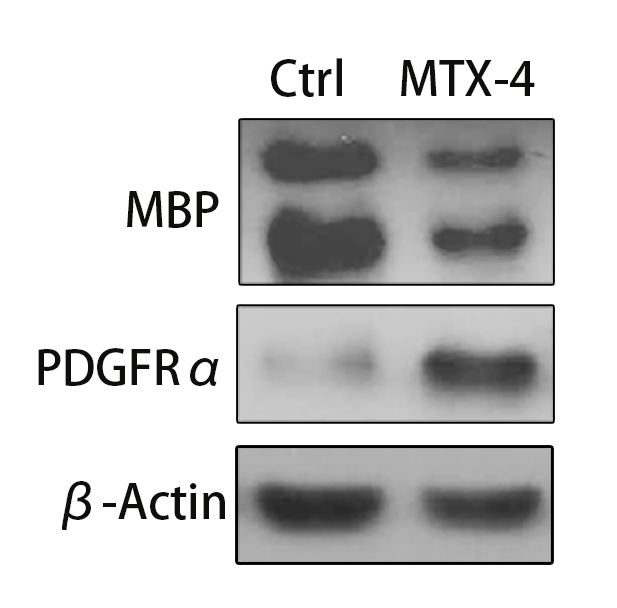


**Figure S3 DHFR inhibition induces oligodendrocyte differentiation defects**

Western blot analysis of MBP (20, 18.5kDa) and PDGFRα (170kDa) protein expression in the spinal cord of control and MTX (4 mg/kg)-treated mice at P15. Beta-actin (43kDa) is the loading control.


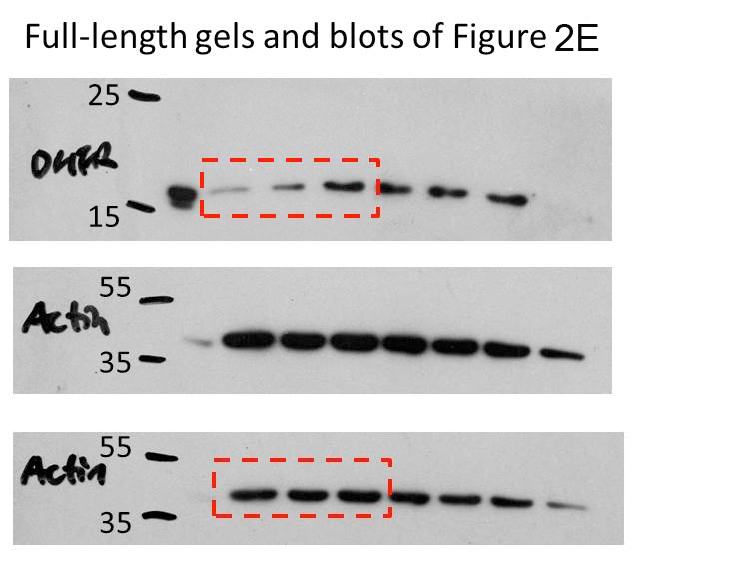


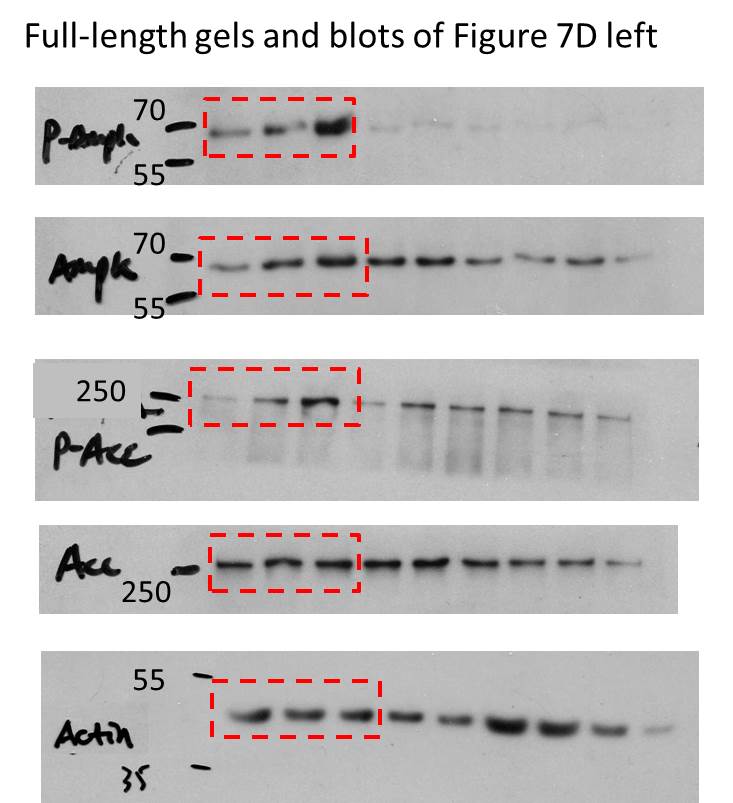


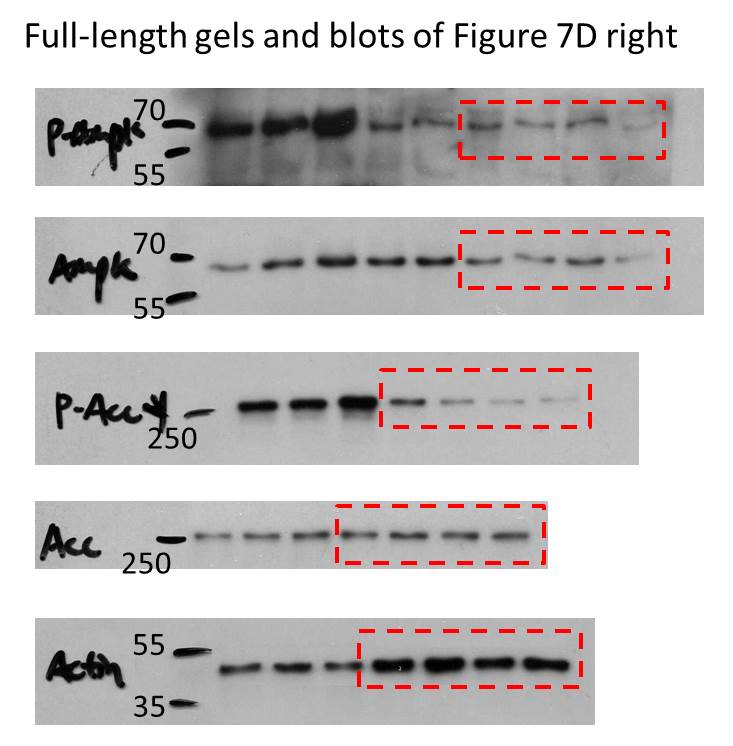


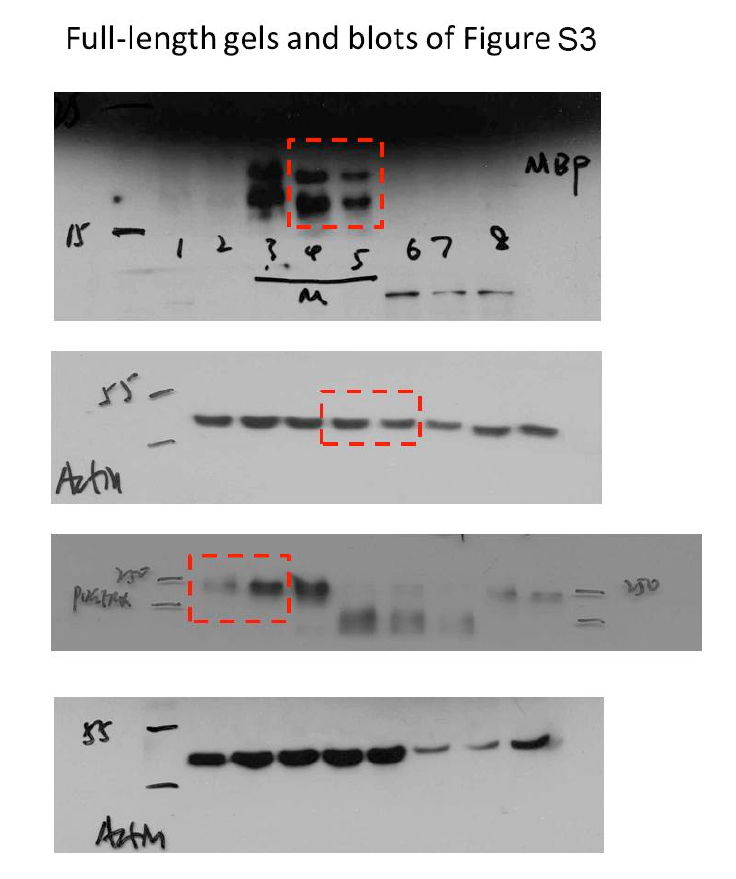

Supplement: Supplementary file 1 — Supplementary data [file 41598_2017_1732_MOESM1_ESM.doc]
